# Preauricular Superficial Temporal Artery Flap: A Simple Solution in Face Reconstruction

**DOI:** 10.7759/cureus.69044

**Published:** 2024-09-09

**Authors:** Gonçalo Tomé, José Miguel Azevedo, Inês Catalão, Dmitry Shelepenko, Carla Diogo

**Affiliations:** 1 Department of Plastic and Reconstructive Surgery and Burns Unit, Coimbra Local Health Unit, Coimbra, PRT

**Keywords:** face, flap, preauricular region, squamous cells carcinoma, superficial temporal artery, superficial temporal artery flap

## Abstract

Face reconstruction is exceptionally demanding for the best color and texture equivalence with minimal morbidity. Most defects result from tumors or trauma, and local flaps are typically used. A preauricular flap based on the superficial temporal artery (STA) is an alternative to the classically used retroauricular flap. We describe a case of an 80-year-old female presenting a frontotemporal squamous cell carcinoma and reconstructed with a preauricular superficial temporal artery (PASTA), reverse-flow, pedicled flap. It measured 5x6 cm and was based on the STA parietal branch instead of the more frequently reported frontal branch. The donor site was closed primarily. After 24 months, there were no complications, the aesthetic result was good, and the scar was inconspicuous. We also reviewed the literature for preauricular flap reports. A total of 152 preauricular STA flaps have been reported, mostly free flaps, for different facial areas, including ear cartilage or hair-bearing areas. Venous congestion is the most frequent complication. The PASTA flap is a simple solution for small-to-moderate facial defects, is easily harvested, has a straightforward dissection, can be reliably based on either a frontal or parietal branch, and provides an excellent texture and color match with minimal morbidity.

## Introduction

Face reconstruction is uniquely demanding, considering the central role that the face possesses in a person’s aesthetics. Soft tissue defects most frequently result from tumor excision and trauma [1]. Non-melanoma skin cancer, the most common worldwide, more frequently affects the head and neck and includes basal cell carcinoma (75-80%) and squamous cell carcinoma (15-20%) [2]. Craniofacial soft tissue trauma injuries are similarly common and represent 7% of emergency department visits [3].

Face reconstructive options range from skin grafts to local, distant, or free flaps. Defect size, specific location, color and texture similarity, adjacent tissue availability, donor site morbidity, and facial aesthetic subunit preservation should guide this decision. Local flaps are typically used as a first-line option for small to moderate-size defects since they offer the best color and texture matching [1]. The preauricular flap based on the superficial temporal artery (STA) is a versatile, interesting, underestimated local option that can reach the entire face, has low morbidity, a concealed scar, and is an alternative to the classically used retroauricular flap. Its pedicle misjudged proximity to the frontal branch of the facial nerve might justify its underuse. Particularly based on the STA frontal branch, it has been recently reported with good results for different facial defects, including chondrocuteneous flap for the eyelid or nasal ala [4].

We intended to describe a case of facial defect reconstruction using the preauricular superficial temporal artery (PASTA), a reverse-flow, pedicled flap based on the parietal branches instead of the more frequently reported frontal branches and analyze the potential of this flap on the coverage of facial small to moderate defects. We additionally reviewed the literature.

## Case presentation

**Figure 1:**
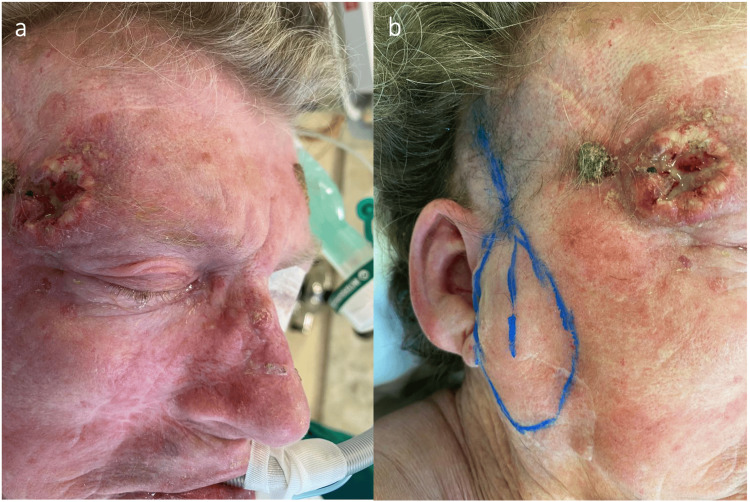
Frontotemporal tumor and flap markings (a) Frontotemporal ulcerated squamous cell carcinoma, (b) preauricular superficial temporal artery flap markings

**Figure 2:**
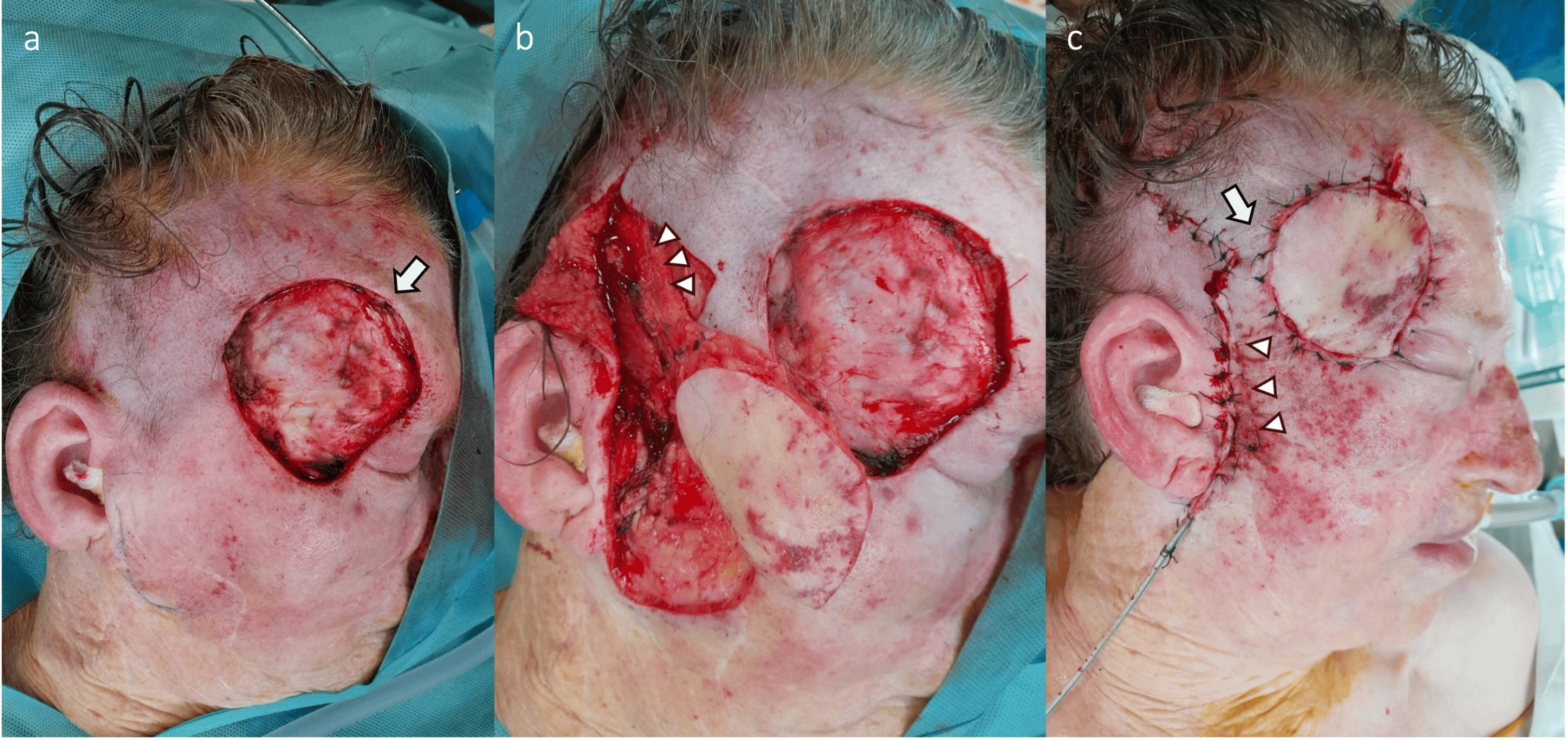
Preauricular superficial temporal artery flap harvest a. Frontotemporal defect after tumor resection (arrow); b. Preauricular superficial temporal artery flap, 5x6 cm, harvested and pedicled on the parietal branch (arrowheads); c. Flap inset with the pedicle subcutaneously tunneled (arrow), and donor-site directly closed (arrowheads)

We present a case of an 80-year-old female patient with an ulcerated cutaneous lesion on her right supraciliary region that had 6 months of evolution. Her previous medical history included other facial skin tumor lesions that were completely excised. She had a pacemaker, auricular fibrillation, and was hypocoagulated with rivaroxaban 20 mg. No cervical or parotid pathological lymph nodes were detected at the presentation. The lesion measured 4 x 5 cm and corresponded to a squamous cell carcinoma (Figure 1). A hand-held doppler was used preoperatively to check and trace the STA and its parietal branch. A resulting frontotemporal defect from tumor complete excision was subsequently covered using an ipsilateral island PASTA flap, based on the parietal branches in a reverse-flow manner, measuring 5 x 6 cm (Figure 2). The flap was inset without tension and sutured with 4/0 vicryl and polypropylene sutures. The donor site was directly closed. The suction drain was left for two days until drainage was lower than 30 cc per day. No immediate complications were observed, and the patient was discharged after 2 days. The other cutaneous lesions she had on her nose corresponded to actinic keratosis and were being treated with topical Imiquimod (Zyclara® 3.5%).

**Figure 3:**
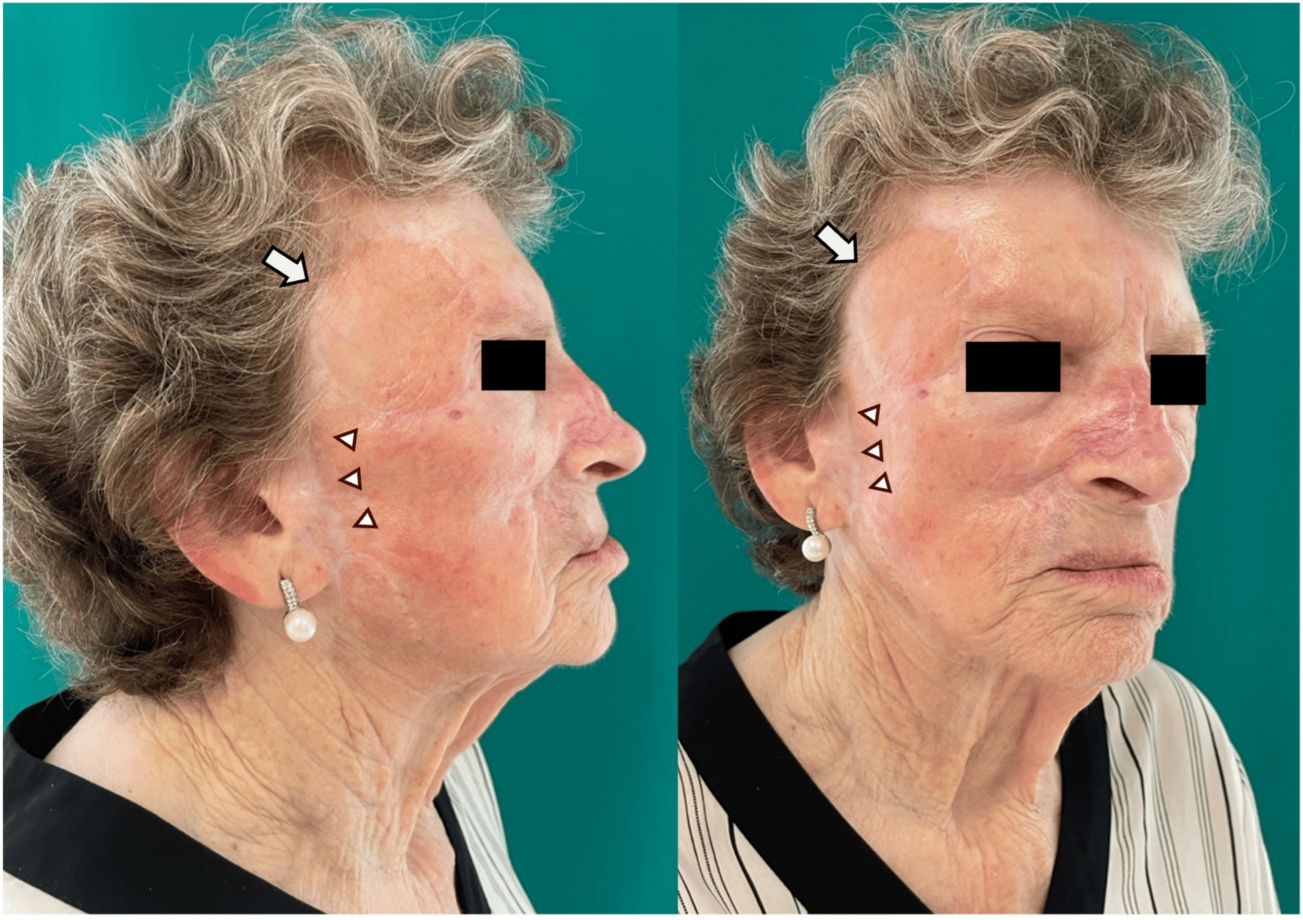
Post-operative result after 24 months Preauricular superficial temporal artery flap (arrows) and donor site (arrowheads), 24 months after the surgery

After 24 months, there were no complications observed pertaining to the flap and donor site, nor was there any significant functional deficit on the face mimic. The aesthetic result was reasonably good on both the reconstructed and donor sites. The flap texture and color similarly matched the area of reconstruction (Figure 3). Scars were inconspicuously concealed in the preauricular area, without any ear distortion. No secondary remodeling or revision surgery was necessary, and no local recurrence of skin tumor was detected during the follow-up.

Vascular anatomy

The STA is a terminal branch of the external carotid artery and generally arises within the parotid gland after giving the maxillary artery. It has a 1.5 mm diameter, a constant course, and can be easily identified through palpation or hand-held Doppler due to its superficial course in the preauricular area, particularly anterior to the tragus [5]. Eye-to-tragus line (ETL), connecting the tragus to the corner of the eye, is a landmark that can be quickly and easily used to locate the STA and its division. STA main branch superficializes from the parotid, usually 1.5 ± 0.45 cm anterior to the tragus on the ETL [6]. STA subsequently bifurcates into the frontal and parietal branches above ETL (93%) [6] or above the zygomatic arch (80%) [5]. These branches are similarly stable and have a good caliber, with the frontal branch having a larger diameter [5,6].

**Figure 4:**
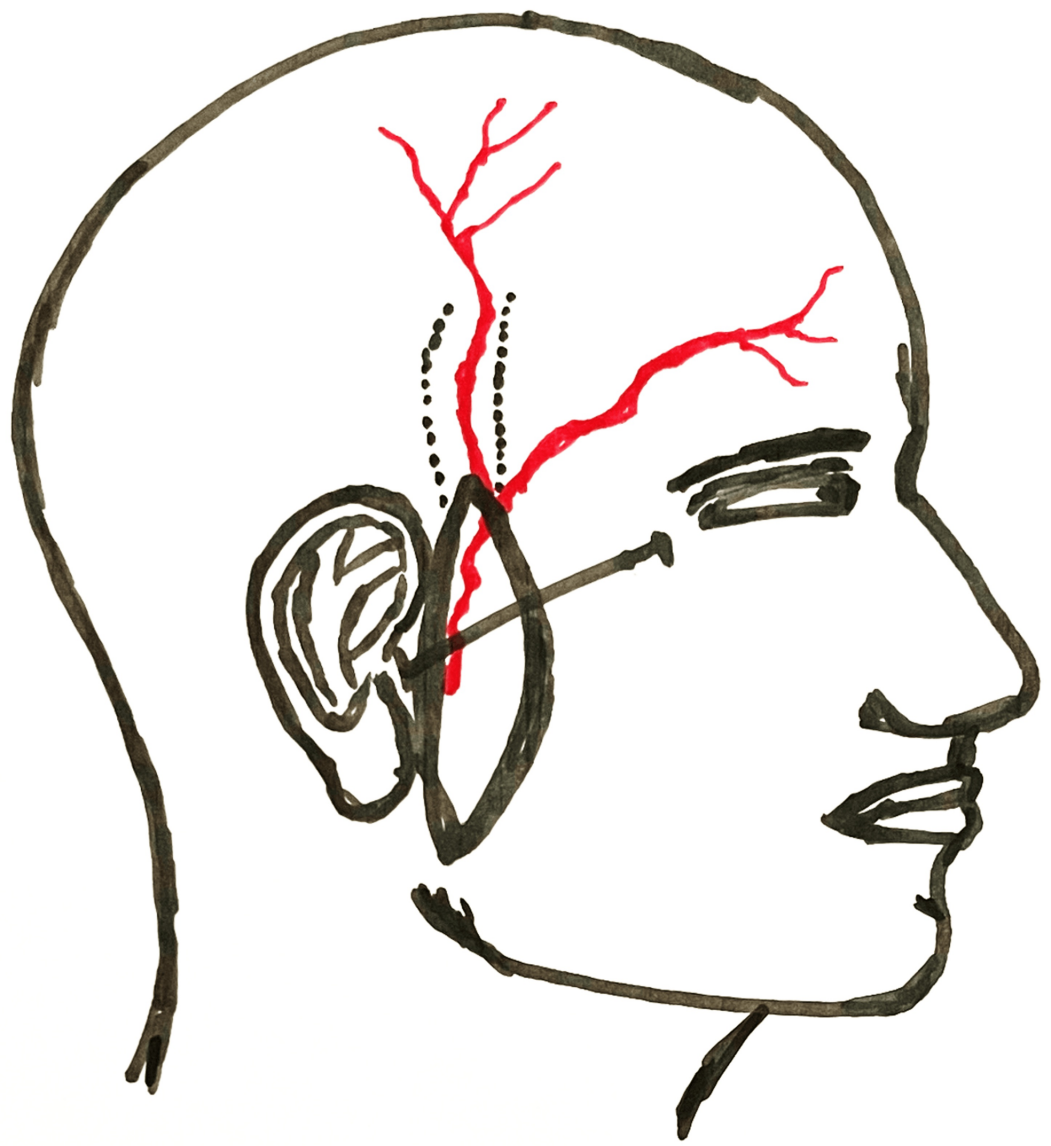
Reverse flow preauricular superficial temporal artery flap, parietal and frontal branches, and the eye-to-tragus line Image credits: Gonçalo Tomé

The rich anastomotic network between STA and the deep temporal artery over the temporal line, with the supraorbital and supratrochlear arteries (frontal branch), and with the occipital artery and contralateral parietal branch (parietal branch), renders the flap to be safely based on the STA in a reverse flow pattern on either branch (Figure 4) [4].

STA runs in the temporoparietal fascia (TPF), which is immediately under the hair follicles in the subcutaneous tissue and continues as the galea aponeurotica above the temporal line and as the superficial muscular aponeurotic system (SMAS) below the zygomatic arch. The superficial temporal vein (STV) frequently does not follow the STA, has a less predictable course, and may remain as one vessel or divide into two to three branches more proximally [4].

Surgical technique

The steps for the pedicled reverse-flow PASTA flap harvest are resumed in Table 1 [4,7]. Preoperatively, a hand-held Doppler is usually used to confirm the integrity and trace the course of the STA and its two main frontal and parietal branches. A computerized tomography angiography is not routinely necessary.

**Table 1:** Surgical steps Adapted from Ausen K [4] and Yamauchi M et al. [7]; ETL: eye-to-tragus line; SMAS: superficial muscular aponeurotic system; STA: superficial temporal artery; STV: superficial temporal vein; TPF: temporoparietal fascia

Number	Main step	Detail
1	Doppler	Hand-held doppler to confirm the integrity and trace the course of STA and its two main frontal and parietal branches.
2	Markings	Preauricular skin island is marked over the STA. Can either include or not hair-bearing area from the sideburns. Eye-to-tragus line (ETL) can help identify STA.
3	Incision, STA and STV identification and ligation inferiorly	Lidocaine 1-2% and adrenaline 1:100.000 association can be used for local infiltration. Skin incision and dissection starting inferiorly and posteriorly, sparing the SMAS and parotid fascia, until identification of STA and STV. STA emergence from the parotid is often 1-2 cm anterior to tragus on the ETL. The vessels are clipped and cut proximally.
4	Flap harvest	Flap harvest from caudal to cranial and including the STA, STV and TPF around the pedicle. Skin incision is extended superiorly, and skin flaps are undermined in the dermohypodermic plane, making vessel identification and dissection straightforward.
5	Unused branch ligation	Dissection until bifurcation, typically above the zygomatic arch. The unused branch is clipped.
6	Pedicle dissection	Continue dissection including TPF with at least 2-3 cm of perivascular tissue around its pedicle to avoid venous congestion and until reaching the pivot point, where it can be transferred without tension. Careful dissection to avoid facial nerve frontal branch damage, which runs just deep to the TPF, and 1-2 cm inferior to the frontal STA branch.
7	Inset	Flap is transferred to the defect through a subcutaneous tunnel. Should be sufficiently wide to avoid pedicle compression.
8	Closure	Primary closure or retroauricular flap donor site closure. A suction drain is left for 2 days or until <30cc a day.

The patient is put in the supine position, prepped, and draped. General anesthesia is administered. The flap can include not a hair-bearing area from the sideburns and helical cartilage, according to the facial area to be reconstructed. The ETL helps identify the STA emergence point, often 1-2 cm anterior to the tragus (Figure 4).

Care should be taken to avoid injuring both hair follicles and the frontal branch of the facial nerve during the dissection of the pedicle, despite the nerve running along the pitanguy line, which is parallel, anterior, and 1-2 cm inferior to the STA frontal branch, and having a relatively safe dissection. Also, the nerve runs under the TPF and vessels [4].

## Discussion

**Figure 5:**
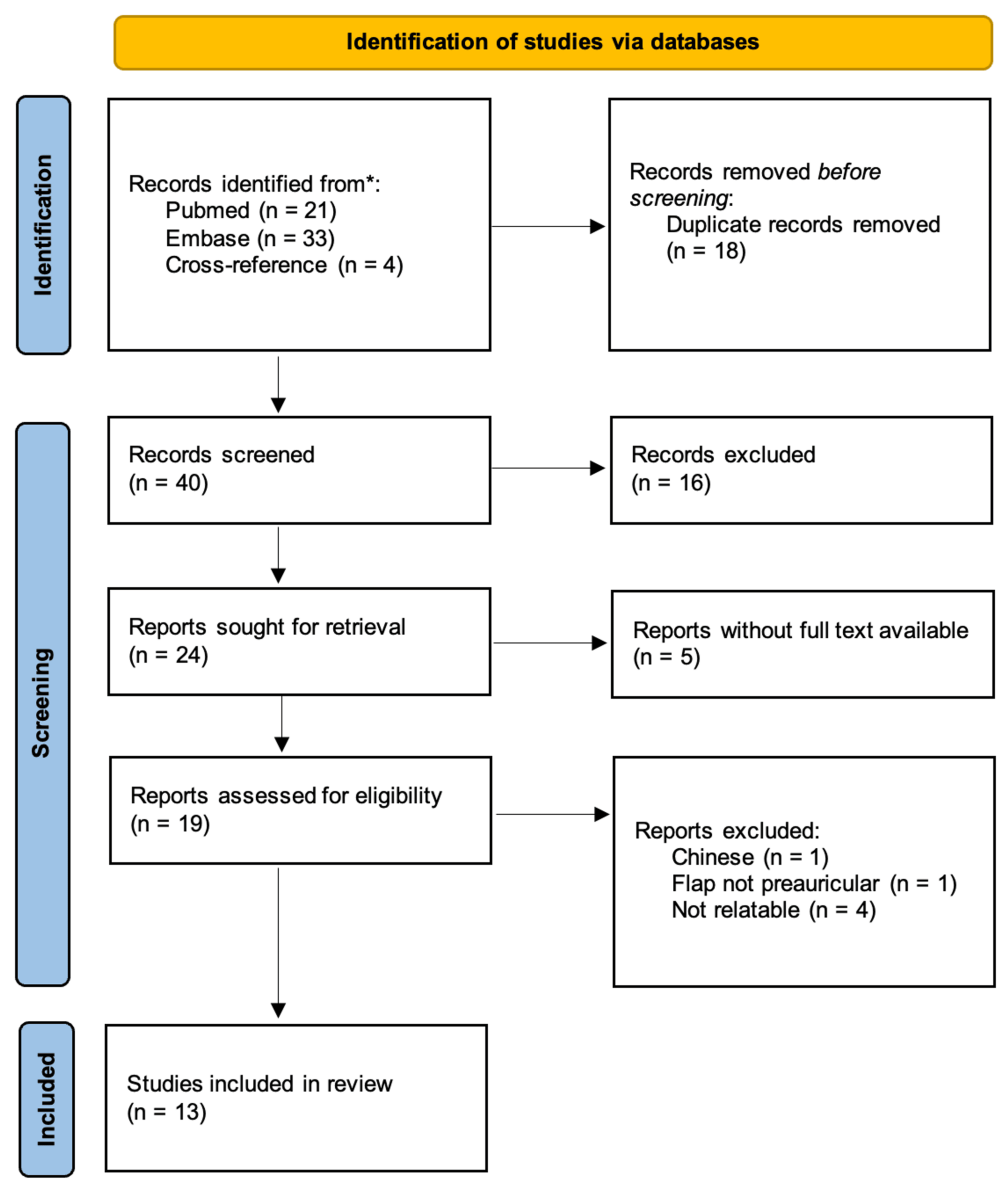
Literature review flow diagram

We did a complementary review of the literature on preauricular flaps based on the STA for facial defect reconstruction. The search was conducted on Pubmed and Embase databases using the terms "preauricular," “superficial temporal artery,” and “flap” for studies published until 30th June 2024. Additional cross-references were included, yielding 58 reports. The records were screened independently by two authors, GT and JMA. During the screening through the title and abstract, we excluded the articles that did not include any detailed case, studies not including PASTA flaps, articles entirely written in languages other than English, Portuguese, or Spanish, and not relatable articles to our study objectives. Papers were subsequently retrieved to be assessed for eligibility. Thirteen records were included (Table 2). Figure 5 resumes the steps that were used during the review, based on PRISMA guidelines.

**Table 2:** Literature review of preauricular flaps based on the STA for face reconstruction Mo: months; STA: superficial temporal artery; STV: superficial temporal vein; y: years

First author and year	Area of reconstruction	Condition	Age (mean, range; years)	Flaps number	Flap	Pedicle	Flap size (mean, range; cm) Pedicle length (cm)	Donor-site	Follow-up (mean, range; years or months)	Complication	Result
Binhimd U et al., 2022 [8]	Concha bowl and external auditory canal	Tumor	40-70	3	Pedicled flap Superficial temporal artery capillary perforator-based island flap	Perforator from STA	Width 2 cm	Closed primarily	4 y	No	Perfectly matched the texture and color of the reconstructed site and the tragal contours were not distorted
Wei J et al., 2020 [9]	Nasal tip	Congenital (n=5), trauma (n=18), tumor (n=2), iatrogenic (n=7)	26.3 (16-56)	32	Free flap Preauricular reversed superficial temporal artery flap	Parietal STA and STV branches	2.5 × 2.0 to 4.0 × 3.6 Pedicle length 5.58 cm artery, 6.21 cm vein	Closed primarily	11 mo (6-79)	Venous congestion and resulting 10% flap necrosis (n=1) Hematoma (n=1)	Excellent functional coverage and improved esthetic appearance Majority (98.5%) rated their outcome as highly improved and improved Secondary debulking (n=22)
Zhao J et al., 2018 [10]	Forehead	Trauma and tumor	37.6 (29–56)	8	Pedicled flap Reversed temporal island flap	Frontal or parietal STA branch	1.5 x 1.5 to 2.2 x 2.8	Closed primarily	15.7 mo (10–24)	No	Scalp incision and donor-site scars hidden by hair and inconspicuous Satisfying result
Oh SJ et al., 2014 [11]	Upper lip (moustache)	Trauma	45	1	Free flap Hairy preauricular free flap	STA and STV	2 x 2.5	Closed primarily	3 y	No	Symmetrical philtral column, hairy beard, and adequate upper lip contour upper lip. Excellent aesthetic appearance Marginal scar revision and debulking
Xu M et al., 2014 [12]	Cheek	Tumor (n= 10), trauma (n=2)	58.7 (46-74)	12	Pedicled flap Pretragal perforator flap	Perforator from STA or posterior auricular artery	4 x 2 to 8 x 3.5 Pedicle length 2,5 cm	Closed primarily	10 mo (6-12)	No	Matching skin quality, donor site scar concealed by the facial contour Satisfied with the aesthetic results
Yamauchi M et al., 2012 [7]	Eyelid (n=4), temporal (n=1), intraoral (n=1)	Tumor (n=4), contracted eye socket (n=2)	42.8 (16-70)	6	Pedicled (n=5) and free (n=1) flap Reverse superficial temporal artery flap from the preauricular region	Frontal or parietal (free flap) STA and STV branches	4.2 x 1.4 to 5.8 x 2.0	Closed primarily (n=4) Retroauricular flap (n=2)	Up to 2 y	Venous congestion (n=1) Temporary facial palsy (n=1)	All cases results were cosmetically favorable and donor-site scar inconspicuous
Zhang YX et al., 2008 [13]	Nose	Trauma/burn (n=31), tumor (n=19), congenital (n=9), iatrogenic (n=4)	15-59	66	Free flap Free vascularized preauricular and helical rim flaps	STA and STV with lateral femoral circumflex vessels interposition grafts	Width 2 cm Pedicle length 10-14 cm	Posterior auricular flap (n=48) Closed primarily (n=18)	Up to 1 y	Flap failure (n=2; 97% success rate)	Functional and aesthetic outcome was satisfactory in the majority Secondary procedures (scar revision and/or pedicle/flap debulking) (n=23)
Li S et al., 2007 [14]	Nasal tip	Trauma	36	1	Free flap Skin Island reverse preauricular superficial temporal artery free flap	STA	3.0 x 2.5 Pedicle length 5 cm	Transposition of a pedicled postauricular flap	1 y	No	Excellent nasal tip contour, color match, and tip projection, and aesthetically pleasing donor area Scar revision flap edges nasal tip
Pascone M et al., 2005 [15]	Lower eyelid	Tumor	50-89	8	Pedicled flap Reverse auricular flap or Chondrocutaneous helix island flap	Frontal STA branch	3 to 4 cm	Closed primarily or Retroauricular flap	1 to 4 y	Partial superficial flap necrosis associated with hematoma and venous congestion (n=1)	Secondary pedicle reduction in subcutaneous tunnel (n=3)
Michlits W et al., 2004 [16]	Nose	Trauma, tumor, iatrogenic	24-55	6	Free flap Chondrocutaneous preauricular (tragus) free flap	STA and STV	Up to 3.5 x 5-6	Closed primarily	6 mo	No	Color and texture similar to surrounding skin Preauricular donor site scar inconspicuous
Bakhach J et al., 1999 [17]	Nose	Trauma (n=6), tumor (n=1)	22-62	7	Pedicled flap Reverse (chondrocutaneous) auricular flap	Frontal STA branch	Length 2-4 cm	Advancement and rotation helical rim flap	Up to 1 y	No	Good to excellent results
Koshima I et al., 1999 [18]	Upper eyelid	Tumor	66	1	Pedicled flap Ear helix flap (reverse flow chondrocutaneous island flap)	Frontal STA branch STV proximal vein supercharged	No information	Closed primarily	4 y	No	Function preserved and donor site with minimal deformity
Parkhouse N et al., 1985 [19]	Nasal ala	Tumor	67	1	Free flap Free chondrocutaneous flap from the ear helix based on the reverse-flow STA	STA and STV	No information	Posterior auricular flap	3 mo	No	Flap remodeling (debulking)

The records consisted of case reports and case series (Table 2). A total of 152 preauricular flaps were reported, mostly consisting of free flaps (n=108, pedicle flaps n=44) [7-19]. Other older reports of preauricular flaps were found but not included because the full text was not available. Binhimd U [8] and Xu M [12] more recently described the use of STA perforator flaps (n=15) for concha bowl/external auditory canal and cheek reconstruction, respectively. The nose was the area more frequently reconstructed, followed by the eyelid, forehead/temporal region, ear, lip, cheek, and oral mucosa. Trauma, tumor resection, and congenital and iatrogenic defects were the causes of facial defects. Most defects and respective flap dimensions were small to moderate, being the largest flap described to have 8 x 3.5 cm. The donor site was either closed primarily or using a retroauricular flap or an advancement-rotation helical rim flap. Venous congestion (n=3) was the main complication, followed by partial flap necrosis (n=2) and free flap failure (n=2). In general, all the flaps presented an excellent color and texture match to the surrounding skin, and the preauricular donor site scar was inconspicuous.

The STA has long been used in pedicled and free flaps for face reconstruction [4]. Its stability, multiple vascular anastomoses, and availability of surrounding tissue enable multiple possibilities for the vast facial areas. It can be used as a scalp, temporal, retroauricular, preauricular, include the helical rim or tragus, be cutaneous, chondrocuteneous, hair-bearing or not, anterograde, retrograde, be based on the frontal or parietal branches, used in a one- or two-stage procedure, perforator, free, or pedicle flap [7-20].

Gradually, the reverse-flow preauricular flap has been refined and proven to provide an excellent texture and color match to many different facial areas [4,7-20]. Compared to the well-known retroauricular flap, the PASTA flap has a better similarity of texture, thickness, and color, being less reddish. Also, its blood supply is better, and the flap is less prone to venous congestion [7,10]. Its retrograde design allows for a longer pedicle, and it can be safely based on either main branch. The frontal branch is more frequently used for pedicle flaps, considering the pivot point's preferable position and wide rotation arc. However, it may leave a donor site that causes a raised ipsilateral eyebrow. Alternatively, the parietal branch is more commonly used when the frontal is not adequate or for free flaps, which can be oppositely dissected from cranial to caudal [4,7,9].

Venous congestion is the most frequent complication, even though we did not observe it in our case [4,7,9,15]. The STV is not so stable, has a variable course, may divide differently, and does not always run with the STA distally [4]. Their distance may be significant enough that STV may not be included in the pedicle, and venous drainage is dependent on small comitant veins around the artery. Thus, the pedicle should importantly include sufficient TPF perivascular tissue, ideally 2 to 3 cm wide [4,7].

In our case, the defect was frontotemporal; the frontal branch was compromised during tumor excision; thus, the parietal branch was chosen and safely used. Pedicle dissection was straightforward, included a 3 cm-wide TPF, and was relatively safe from the frontal branch of the facial nerve. This nerve often runs deep to TPF, parallel, and inferior (1-2 cm) to the frontal STA branch. Though safe dissection, great care must always be taken during the procedure [4,7]. The flap was easily harvested, and pleasantly thin with excellent surrounding tissue matching and aesthetic results. Donor-site left no ear or sideburn deformity and an unsightly scar. To the best of our knowledge, this has been the largest PASTA flap described (5x6 cm; 30 cm2), with donor-site direct closure. This report emphasizes this flap's aesthetic and functional effectiveness, providing good color and texture match with minimal morbidity, prompting its incremental use for face reconstruction.

## Conclusions

The reverse-flow pedicle PASTA flap is an excellent and simple solution for small to moderate defects in the different areas of the face. The pedicle is reliable and constant, has a straightforward and safe dissection, and can be based on either frontal or parietal STA branches. It can include helix or tragus cartilage or a hair-bearing area, be used as a pedicled or free flap, provide a harmonic color and texture match, and provide a good aesthetic result with minimal donor-site morbidity.

## References

[1] Wusiman P, Tuerxun J, Ling W (2016). Middle and lower face soft tissue reconstruction: a 10-year retrospective study. Indian J Otolaryngol Head Neck Surg.

[2] Kansara S, Bell D, Weber R (2020). Surgical management of non melanoma skin cancer of the head and neck. Oral Oncol.

[3] Braun TL, Maricevich RS (2017). Soft tissue management in facial trauma. Semin Plast Surg.

[4] Ausen K, Pavlovic I (2011). Flaps pedicled on the superficial temporal artery and vein in facial reconstruction: a versatile option with a venous pitfall. J Plast Surg Hand Surg.

[5] Koziej M, Wnuk J, Polak J (2020). The superficial temporal artery: A meta-analysis of its prevalence and morphology. Clin Anat.

[6] Jean-Philippe H, Benoît B, Françoise K, Michael D (2021). Anatomy and external landmarks of the superficial temporal artery using 3-dimensional computed tomography. Surg Radiol Anat.

[7] Yamauchi M, Yotsuyanagi T, Yamashita K, Ikeda K, Urushidate S, Mikami M (2012). The reverse superficial temporal artery flap from the preauricular region, for the small facial defects. J Plast Reconstr Aesthet Surg.

[8] Binhimd U, Alkaabi SA, Alsabri GA, Honart JF, Leymarie N, Kolb F (2022). Superficial temporal artery capillary perforator-based island flap for conchal bowl and external auditory canal reconstruction. Ann Chir Plast Esthet.

[9] Wei J, Chen Q, Herrler T, Xu H, Li Q, He J, Dai C (2020). Supermicrosurgical reconstruction of nasal tip defects using the preauricular reversed superficial temporal artery flap. J Plast Reconstr Aesthet Surg.

[10] Zhao J, Song G, Zong X (2018). Using the reversed temporal island flap to cover small forehead defects from titanium mesh exposure after cranial reconstruction. World Neurosurg.

[11] Oh SJ (2014). Aesthetic reconstruction of a superficial defect of the upper lip using a hairy preauricular free flap. J Craniofac Surg.

[12] Xu M, Yang C, Li JH, Lü WL, Xing X (2014). Reconstruction of the zygomatic cheek defects using a flap based on the pretragal perforator of the superficial temporal artery. J Plast Reconstr Aesthet Surg.

[13] Zhang YX, Yang J, Wang D (2008). Extended applications of vascularized preauricular and helical rim flaps in reconstruction of nasal defects. Plast Reconstr Surg.

[14] Li S, Wang J, Cao W, Yin C, Chang TS (2007). A free reverse preauricular flap for reconstruction of a nasal defect. Plast Reconstr Surg.

[15] Pascone M, Papa G (2005). The reverse auricular flap for the reconstruction of extended defects of the lower eyelid. Br J Plast Surg.

[16] Michlits W, Papp C, Hörmann M, Aharinejad S (2004). Nose reconstruction by chondrocutaneous preauricular free flaps: anatomical basis and clinical results. Plast Reconstr Surg.

[17] Bakhach J, Conde A, Demiri E, Baudet J (1999). The reverse auricular flap: a new flap for nose reconstruction. Plast Reconstr Surg.

[18] Koshima I, Urushibara K, Okuyama H, Moriguchi T (1999). Ear helix flap for reconstruction of total loss of the upper eyelid. Br J Plast Surg.

[19] Parkhouse N, Evans D (1985). Reconstruction of the ala of the nose using a composite free flap from the pinna. Br J Plast Surg.

[20] Fatma B, Alper U, Mehmet B (2019). The superficial temporal artery island flap: an option for moustache reconstruction. J Craniofac Surg.

